# Optimization of somatic cell injection in the perspective of nuclear transfer in goldfish

**DOI:** 10.1186/1471-213X-10-64

**Published:** 2010-06-08

**Authors:** Pierre-Yves Le Bail, Alexandra Depince, Nathalie Chenais, Sophie Mahe, Gerard Maisse, Catherine Labbe

**Affiliations:** 1 INRA, Cryopreservation and Regeneration of Fish, UR1037 SCRIBE, Campus de Beaulieu, F-35 000 Rennes, France; 2 AFSSA-LEREPP, Unité de Virologie Immunologie Porcines, Ploufragan, France

## Abstract

**Background:**

Nuclear transfer has the potential to become one strategy for fish genetic resources management, by allowing fish reconstruction from cryopreserved somatic cells. Survival rates after nuclear transfer are still low however. The part played by unsuitable handling conditions is often questioned, but the different steps in the procedure are difficult to address separately. In this work led on goldfish (*Carassius auratus*), the step of somatic cells injection was explored. Non-enucleated metaphase II oocytes were used as a template to explore the toxicity of the injection medium, to estimate the best location where the cell should be injected, and to assess the delay necessary between cell injection and oocyte activation.

**Results:**

Trout coelomic fluid was the most suitable medium to maintain freshly spawned oocytes at the metaphase II stage during oocyte manipulation. Oocytes were then injected with several media to test their toxicity on embryo development after fertilization. Trout coelomic fluid was the least toxic medium after injection, and the smallest injected volume (10 pL) allowed the same hatching rates as the non injected controls (84.8% ± 23). In somatic cell transfer experiments using non enucleated metaphase II oocytes as recipient, cell plasma membrane was ruptured within one minute after injection. Cell injection at the top of the animal pole in the oocyte allowed higher development rates than cell injection deeper within the oocyte (respectively 59% and 23% at mid-blastula stage). Embryo development rates were also higher when oocyte activation was delayed for 30 min after cell injection than when activation was induced without delay (respectively 72% and 48% at mid-blastula stage).

**Conclusions:**

The best ability of goldfish oocytes to sustain embryo development was obtained when the carrier medium was trout coelomic fluid, when the cell was injected close to the animal pole, and when oocyte activation was induced 30 min after somatic cell injection. Although the experiments were not designed to produce characterized clones, application of these parameters to somatic cell nuclear transfer experiments in enucleated metaphase II oocytes is expected to improve the quality of the reconstructed embryos.

## Background

When somatic cells are cryobanked for preservation of valuable genetic resources, somatic cell nuclear transfer is the only technology which can subsequently be used to sustain fish reconstruction. Somatic cells hold both paternal and maternal genome and their fitness towards cryobanking [1,2] compensates for the inability of oocytes and whole embryo to withstand cryopreservation [3]. Besides, fish ability regarding cross-species nuclear transfer [4] is expected to facilitate reconstruction of rare individuals with eggs from easily farmed species. Nuclear transfer in fish was developed using embryonic cells [4-8] and more differentiated cells including somatic cells [9-12] as nucleus donor. Up to recently however, nuclear transfer in fish was developed only on activated eggs and on eggs which were activated at the onset of nucleus injection [13]. One reason is that for most studied species, egg activation is spontaneously induced either by oocyte dilution in artificial media (cyprinids) or by egg pricking (medaka). In these species as in amphibians, the first mitosis is initiated in the first thirty minutes after fertilization and meiosis resumption. Therefore, nuclear transfer in activated eggs where maturation/mitosis promoting factor (MPF) levels decrease rapidly [14] raises the question of the quality of nuclear reprogramming. It is known in mammals that nuclear transfer outcome is improved when the injected nucleus is incubated into the recipient oocyte several hours prior to activation. The extent to which nucleus incubation in oocyte cytoplasm prior to activation is important for the success of nuclear transfer was only recently addressed in zebrafish [15] and such issue deserves special attention in rapidly developing fish species.

Whatever the species considered for nuclear transfer, donor nucleus is introduced into the recipient oocyte either by electrofusion or by intracytoplasmic injection. Electrofusion is widely used in several mammals (bovine [16], pig [17], sheep [18], goat [19]), but intracytoplasmic injection is preferred in some species (horse [20], and mice [21]). In fish, the oocytes are so much bigger than the donor cell that electrofusion was barely attempted [7] and most groups use intracytoplasmic injections [6,8-10,13,15,22-24]. Contrarily to fusion, nuclear transfer by intracytoplasmic injection is the procedure the most different from fertilization, but the conditions the most suitable for the resulting embryo development were little explored in vertebrates. Among important factors, the carrier medium may interfere with the subtle cytoplasmic biochemical equilibrium, and the location at which the nucleus is injected inside the highly polarized oocyte [25] may influence chromatin exposure to the required cytoplasmic factors. One reason for such little information in mammals may lay in the difficulty to get enough oocytes of comparable quality which could be used to test several injection conditions in comparable environment. Besides, the survey of many embryo developments after transplantation requires large and costly facilities. Last, maternal effect via placental exchanges is another difficulty to accurately evaluate the consequence of early treatments on development [26]. Such difficulties are not present when nuclear transfer is performed in fish. In goldfish *Carassius auratus*, females spawn thousands oocytes at the same time, the quality within spawns is homogeneous and can be assessed easily, and embryos develop in water without maternal exchanges. The issue of the injection procedure is therefore much easier to analyze in this species than in mammals.

The objective of this study was to characterize and control the parameters the most likely to interfere with the success of nuclear transfer in fish. We first investigated the conditions which allowed goldfish oocyte manipulation without activation induction. Specific media formulated for carp, zebrafish and goldfish oocyte handling during androgenesis and short term storage were tested. Second, we explored the donor cell injection procedure in oocytes. The media which can be used for cell injection and the injected volume were tested for their toxicity after oocyte fertilization. Nuclear transfer experiments were led with fin cells to test whether the injection depth and the incubation time before oocyte activation in water could affect embryo development. Last, cells injected as a whole were monitored within the oocyte to assess plasma membrane rupture. In these nuclear transfer experiments, oocytes were not enucleated. Although the development rates of the embryos were used to estimate the suitability of the injection procedure, the experiments were not designed for clone production. This is why no genetic analysis of the produced embryos was undergone, and no clone production was claimed from our results.

## Results

### Selection of the medium preventing oocyte activation

**Figure 1:**
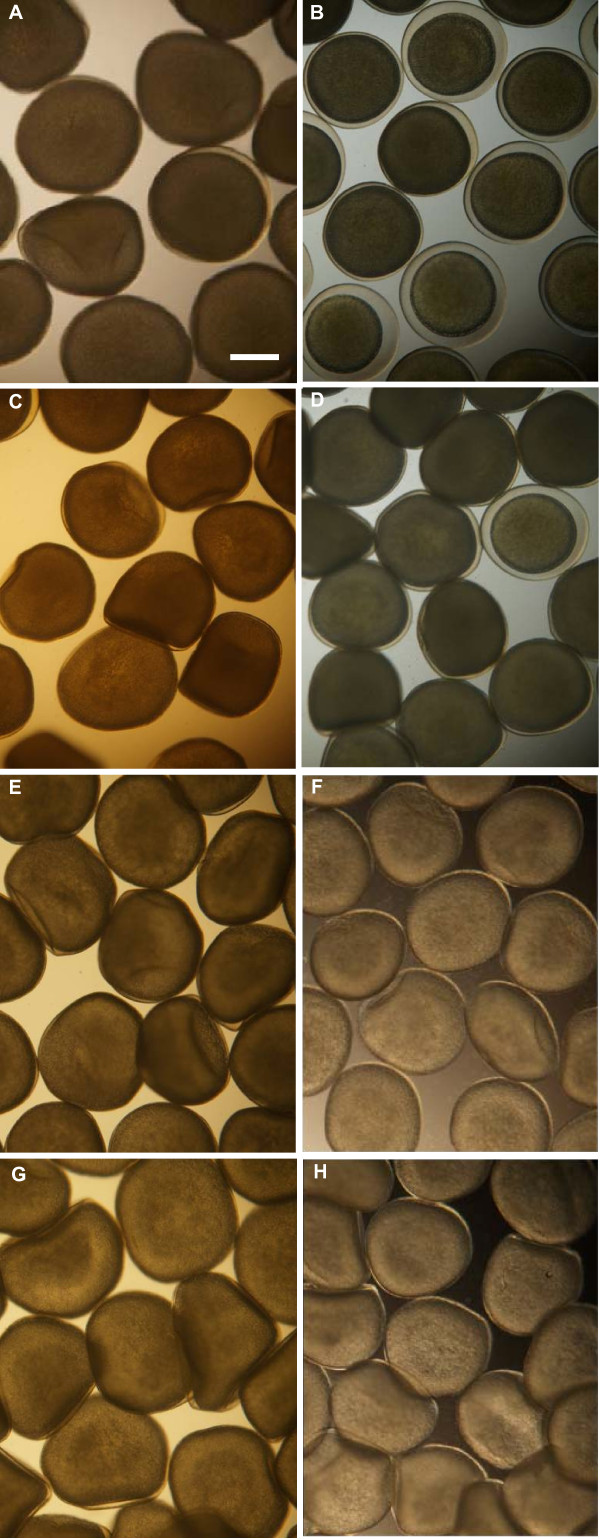
**Assessment of spontaneous cortical reaction of oocytes incubated in different media**. Goldfish ringer after 5 (A) and 25 min (B) incubation; Synthetic ovarian fluid after 5 (C) and 25 min (D) incubation; goldfish ringer with 0.1 mg/ml STI after 5 (E) and 25 min (F) incubation; trout coelomic fluid after 5 (G) and 25 min (H) incubation. Cortical reaction is visualized by the thin transparent layer appearing around the oocyte. Scale bar = 500 μm.

**Figure 2:**
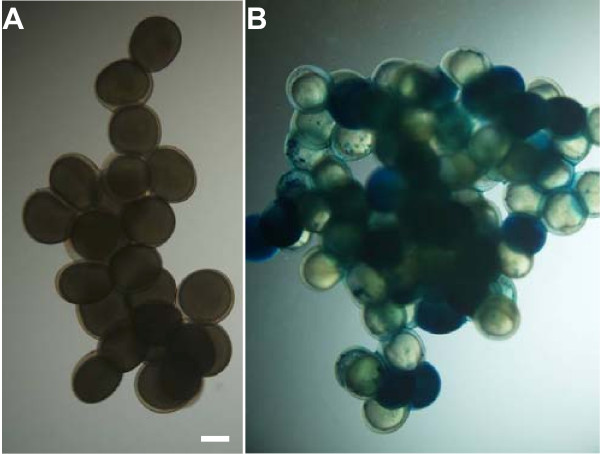
**Aggregation of fertilized eggs incubated in SOF with STI prior to activation**. Oocytes were incubated for 30 min in soybean trypsin inhibitor (STI) 0.1 mg/mL in synthetic ovarian fluid (SOF) prior to fertilization. The picture was taken 5 min (A) and 24 h (B) after fertilization. Egg aggregation impaired embryo development as seen at 24 h by the darkening of dead embryos, although live embryos (translucent) are still visible. Scale bar = 500 μm.

When freshly spawned oocytes were incubated in synthetic media, either goldfish Ringer (GFR) or synthetic ovarian fluid (SOF), they underwent a spontaneous cortical reaction which was slightly slower in SOF than in GFR (Figure 1). Both samples were thereafter unsuitable for fertilization (table 1). When soybean trypsin inhibitor (STI) was added to SOF, activation was prevented but eggs underwent a massive aggregation upon fertilization (Figure 2). These clusters induced developmental problems likely because of oxygen deprivation or nitrate poisoning, and only few embryos per batch could hatch normally. Oocyte incubation in GFR with STI yielded a good protection against activation (Figure 1) and more than 60% development at 24 h were achieved (Table 1). All the concentration tested, from 0.1 to 1 mg/mL, helped to prevent oocyte activation although 1 mg/mL STI induced a slight toxicity as shown from the reduced development rates at 24 h stage. Addition of bovine serum albumin (BSA) to STI did not further improve oocyte inactivated state, and some aggregation upon activation also occurred with this medium. Trout coelomic fluid (TCF) was by all mean the best inactivation medium (Figure 1). Subsequent activation during fertilization did not yield any aggregation, and development rates were the highest among all media tested (Table 1). Development rates above 100% at 24 h and at hatching indicated that incubation in TCF sustained oocyte quality even better than when oocytes were kept into their spawning liquid (controls). Incubation in TCF for up to 1 hour yielded the same development quality (not shown).

**Table 1:** Embryo development after oocyte incubation in different media prior to fertilization.

		Embryo development (%)	
		
Incubation medium (30 min 20°C)	Spawn number	24 h stage	Hatching
SOF	6	3.9 ± 3.2 (a)	0 (a)
GFR	5	3.2 ± 3.0 (a)	0 (a)
+ STI 0.1 mg/mL	10	62.0 ± 15.3 (b)	26.6 ± 8.4 (b)
+ STI 0,25 mg/mL	6	62.3 ± 18.3 (b)	26.6 ± 7.0 (b)
+ STI 0,5 mg/mL	4	44.3 ± 10.0 (b)	25.7 ± 12.9 (b)
+ STI 1 mg/mL	4	29.2 ± 18.9 (c)	19.8 ± 12.4 (b)
+ STI - BSA 0.5%	6	41.9 ± 28.9 (b, c)	nd
TCF	8	104.4 ± 6.1 (d)	105.8 ± 18.5 (d)

Developments after fertilization are expressed as a percentage of the control oocytes in the same spawn at the same stage (kept in spawning fluid for 30 min) (mean ± SE); 150 to 200 oocytes were counted in each sample. SOF synthetic ovarian fluid, GFR goldfish ringer, STI soybean trypsin inhibitor in GFR, TCF trout coelomic fluid, nd: not determined (see text). Different letters within rows indicate significant differences (p < 0.05).

### Suitability of the injected medium toward embryo development

We first observed that oocytes which were pricked but in which no medium was injected kept their ability to be fertilized and to sustain embryo development (90% hatching rate, Table 2). Development rates after pricking were not different from those of the spawn quality control (p > 0.05). Plasma membrane rupture and penetration of the micro capillary were therefore not deleterious to the oocytes. When 50 μL phosphate buffered saline (PBS) or culture medium were injected after micro capillary penetration into non activated oocytes, a decrease in development rates after fertilization was observed at all embryonic stages, and hatching rates were low (13-14%, Table 2). TCF was the least toxic medium although hatching rates were reduced (60%) compared to non injected oocytes. Interestingly, addition of antibiotics to TCF had no further effect on embryo viability (57% hatching rate). Although goldfish coelomic fluid (GCF) injection allowed the same early development rates as did TCF, hatching rates were much reduced in the GCF injected oocytes (13%).

**Table 2:** Embryo development after medium injection into non activated oocytes prior to fertilization.

		Embryo development (%)		
		
Injected medium (50 pL)	Spawn number	Mid-blastula	24 h stage	Hatching
Pricked (no injection)	4	97.1 ± 3.6 (a)	93.5 ± 4.7 (a)	89.7 ± 12.2 (a)
PBS	3	27.4 ± 4.2 (b)	25.7 ± 9.5 (b)	14.1 ± 7.7 (b)
Culture medium	3	56.0 ± 21.5 (b)	42.0 ± 28.0 (b)	13.0 ± 22.5 (b)
SOF	3	68.0 ± 18.3 (b)	52.7 ± 20.2 (b)	14.3 ± 5.0 (b)
TCF	6	87.5 ± 5.6 (c)	76.7 ± 12.8 (b)	60.4 ± 16.6 (c)
GCF	4	79.0 ± 15.4 (b, c)	74.8 ± 14.4 (b)	13.0 ± 10.4 (b)
TCF + antibiotics	6	80.6 ± 10.5 (c)	73.0 ± 19.9 (b)	57.0 ± 23.2 (c)

Developments after fertilization are expressed as a percentage of the control oocytes (not injected) in the same spawn at the same stage (mean ± SE); 25 to 50 oocytes were used in each treatment for one spawn. PBS phosphate buffer solution, SOF synthetic ovarian fluid, TCF trout coelomic fluid, GCF goldfish coelomic fluid. Different letters within rows indicate significant differences (p < 0.05).

The observed reduction in hatching rates whatever the injected medium led us to suspect that embryo disturbance might be due to the volume added to the oocyte. We therefore tested whether a smaller injected volume of TCF would be less deleterious. For the smallest volume tested (10 pL), the outward liquid displacement inside the tip of the micro capillary corresponded to 4 fold the cell size. Such small volume was still enough to allow cell injection in the later on nuclear transfer experiments. For the largest volume tested (50 pL), the outward liquid displacement inside the tip of the micro capillary was about 0.25 mm. Injection of the smallest volume of TCF improved the embryo development rates after fertilization when compared to development rates of oocytes which received the largest volume (Table 3). Hatching rates of the 10 pL injected oocytes (84.8% ± 23.1) were not significantly different from those of the spawn quality controls.

**Table 3:** Embryo development in relation to the TCF volume injected into the oocytes prior to fertilization.

		Embryo development (%)		
		
Injected volume	Spawn number	Mid-blastula	24 h stage	Hatching
50 pL	7	76.8 ± 12.4 (a)	67.1 ± 16.6 (a)	50.4 ± 20.3 (a)
10 pL	4	90.6 ± 8.0 (b)	83.2 ± 16.0 (b)	84.8 ± 23.1 (b)

Developments after fertilization are expressed as a percentage of the control oocytes in the same spawn at the same stage (mean ± SE); 25 to 28 oocytes were tested in each sample. TCF trout coelomic fluid. Different letters within rows indicate significant differences (p < 0.05).

### Importance of the cell injection location during nuclear transfer

**Figure 3:**
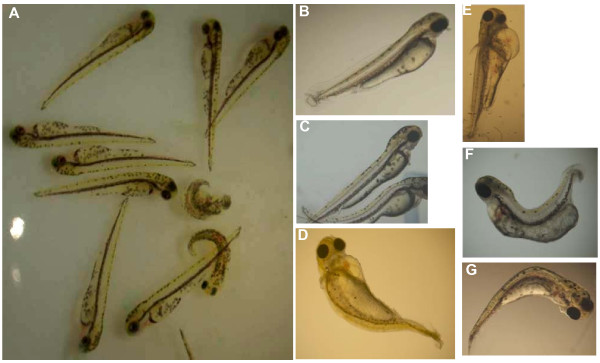
**Morphology of reconstructed fries at hatching**. A: Control fries after fertilization. B, C: reconstructed fries with normal morphology; D, E: reconstructed fries with a large cardiac cavity; F, G: reconstructed fries with skeleton curvature.

When non activated oocytes were injected with TCF only, no parthenogenetic development was observed after activation (n = 40 oocytes from 2 spawns). Eggs underwent cortical reaction and blastodisc formation, but no embryo development was induced. It is only when a somatic cell was injected that some development occurred after oocyte activation. Whatever the injection depth, some developments were observed up to the mid-blastula stage in every spawn, but the shallow injected cells yielded much higher development rates than did the deep injected ones (59% and 23% respectively at the mid-blastula stage, Table 4). Reconstructed embryos from the shallow injected samples were the only ones to develop up to 24 h and hatching. Hatched fries from the shallow samples had however thoroughly altered morphologies and only 4 fries (out of the 40 eggs × 7 spawns) developed normally. Some nuclear transferred embryos had a reduced ability to digest the chorion and had to be mechanically helped for hatching. Examples of fry morphology at hatching are shown Figure 3. Some fries were not different from the fertilized controls; others had a huge cardiac cavity or a bent skeleton. The reason for fry abnormalities and the fry genetic origin were not explored in the present work.

**Table 4:** Development of reconstructed embryo in relation to the cell injection location.

	Embryo development (%)							
	
	Mid-blastula		24 h		Hatching stage		Normal morphology at hatching	
				
Cell location	Mean	Min - max	Mean	Min - max	Mean	Min - max	Mean	Min - max
Shallow	59.3 ± 5.9 (a)	50 - 70	16.5 ± 8.6 (a)	5 - 30	14.5 ± 7.0 (a)	7 - 23	2.0 ± 2.1 (a)	0 - 5.0
Deep	22.9 ± 4.7 (b)	15 - 30	0.0 ± 0 (b)	-	0.0 ± 0 (b)	-	0.0 ± 0 (b)	-

Developments after oocyte activation are expressed as a percentage of the spawn quality control in the same spawn at the same stage (mean ± SE, n = 7 spawns); 40 oocytes were tested in each nuclear transferred sample. Injected oocytes were incubated for 30 min prior to activation.

### Effect of cell incubation time prior to oocyte activation

**Figure 4:**
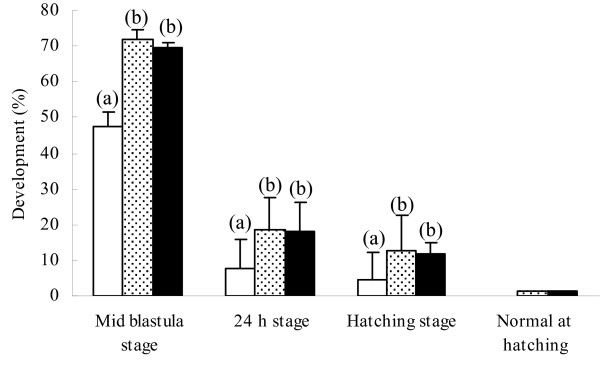
**Development of reconstructed embryo in relation to the incubation time prior to oocyte activation**. Developments are expressed as a percentage of the spawn quality controls in the same spawn at the same stage (mean ± SE, n = 4 to 6 spawns). Forty oocytes were tested in each spawn. Cells in 10 pL TCF were introduced in the shallow position and oocytes were activated after 0 min (white bars), 30 min (dotted bar), and 60 min (black bar). (a) (b): Different letters within one stage indicate significant differences (p < 0.05).

After somatic cell injection, oocyte activation was delayed for up to 1 h. When cell incubation prior to oocyte activation was reduced to less than 1 min, development rates of the reconstructed embryos were low (48% at mid-blastula stage, Figure 4), and none of the hatched embryos had a normal morphology. Increasing the cell incubation time from 0 to 30 min improved development rates at all stages (72% at mid-blastula stage). A 60 min incubation time prior to activation did not further improve development rates compared to 30 min, although the variability of hatching rates was slightly reduced. When development rates at 24 h and at hatching were expressed as a percentage of the corresponding embryo number at mid-blastula, the values were higher for the 30 min embryos (26% ± 14) than they were for the 0 min ones (17% ± 6).

**Figure 5:**
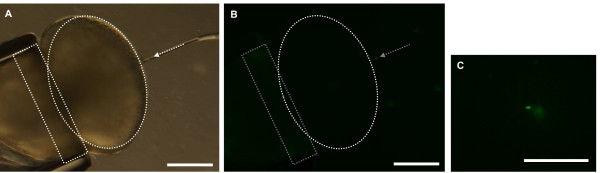
**Rupture of fin cell plasma membrane upon injection**. A: bright field view showing an intact cell at the tip of the capillary (grey arrow). B: Same view as A under fluorescent light, see the green dot of calcein entrapped into the cell cytoplasm. C: fluorescent view few seconds after cell injection. Four cells were injected in order to get a better representation of the different cases. Two cells were still intact: see the two bright spots of calcein still entrapped into cell cytoplasm, whereas the 2 others had a ruptured membrane: calcein was released and its green fluorescence diffused in the surroundings. Scale bar = 500 μm.

Because fin cell plasma membrane was difficult to tear open, whole cells were injected into the oocytes. Membrane rupture was assessed through the dilution of a cytoplasmic fluorescent dye entrapped into the intact somatic cells. When somatic cell cytoplasm was labeled with calcein acetoxymethylester (Calcein AM), cells appeared as a bright green spot (Figure 5). In the first minute after injection, this bright spot faded into a green diluted signal. Signal dilution was caused by plasma membrane rupture and intracellular calcein release within the oocyte cytoplasm. The time between cell injection and membrane rupture varied between 12 and 45 s, with an average of 25.1 s ± 11.8 (n = 15). When the same batch of labeled cells was aspirated and released into TCF instead of being released into the oocyte, the bright spots stayed intense for several hours (n = 15). This indicated that membrane rupture was caused by cell exposure to oocyte cytoplasm and not by the mechanical challenge due to the aspiration-injection process.

## Discussion

### Prevention of oocyte activation

In the perspective of nuclear transfer in metaphase II oocytes, incubation conditions must maintain oocyte quality during the whole injection process. Accidental activation will trigger MPF degradation [14] and the subsequent environment in which the somatic nucleus is exposed will be less favorable to reprogramming. Our data confirmed that synthetic media with STI prevented goldfish oocyte activation [27], but none of the tested synthetic media was as efficient as TCF at maintaining oocyte quality during incubation. This is in accordance with the recent achievement of nuclear transfer in zebrafish using Chinook salmon coelomic fluid [15]. It is difficult to decipher from the current knowledge which TCF component is responsible for this inactivation property. TCF is known to contain heat and acid stable serine protease inhibitor activity [28], high glucose concentration (2 mM) and a high pH (8.4-8.8) [29]. However, all our attempts to mimic these features with artificial media failed to reach TCF performance. Some bacteriostatic activity was also described [30]. Another favorable component might be TCF hormonal content as [31] demonstrated that progesterone contributed to oocyte quality in goldfish.

### Choice of the injection medium

We used the fertilization test to determine which injection medium was the least toxic for the oocyte and the subsequent embryo development. Several media were tested on spawn replicates, and this approach was easier than it would have been in nuclear transfer experiments. We therefore assumed that the medium injected into the oocyte interfered in the fertilized eggs according to the same pattern as it would have done in nuclear transferred zygotes. TCF was the least toxic medium for embryo development after injection into the oocytes. Surprisingly, injection of GCF reduced hatching rates, and we suspect that it is because the quality of the collected GCF was not as good as that of the TCF. Indeed, goldfish spawns contain little GCF unless they are collected at least 5 hours after ovulation [32]. At this time, spawns are already ageing, and it is likely that GSF quality is reduced as a consequence. On the contrary, spawn ageing is much slower in trout [33]. This may explain the best fitness of TCF over GCF in the injection experiments. We do no know why all three synthetic media were toxic for embryo development. We can only suspect that some components such as phosphate and calcium chloride interfered with the oocyte endogenous calcium whose concentrations are so finely regulated during activation [34,35] and mitosis [36].

### Plasma membrane rupture after injection

We do not know what made fin cultured cells so difficult to disrupt prior to injection. The excess of plasma membrane allowing cell movement in culture is one reason for plasma membrane plasticity and deformability. To circumvent this problem, [15] used piezo pulses to induce plasma membrane rupture. Our results showed that oocyte cytoplasmic factors had the potential to readily induce membrane rupture after injection, although the involved mechanisms are unclear. In oocyte, the rapid disassembly of sperm nuclear membrane results from the disruption of the lamina scaffold [37,38]. Plasma membrane cytoskeleton may have been sensitive to a similar process within the oocyte.

### Non enucleated oocyte used as recipient

In this work, the injection procedure was optimized on non enucleated oocytes, in an attempt to reduce the development rate variability which would have been randomly induced during enucleation. Indeed, blind aspiration of the female pronucleus after activation [9,11,39] induces the loss of cytoplasmic factors, and oocyte irradiation [5,40] alters oocyte proteins and maternal mRNAs. Recently, [15] used laser irradiation, a promising method which had never been used before in fish, but whose effects on oocyte quality are unknown. Since our experiments were designed to explore the injection procedure, we did not undergo genetic analysis of the produced embryos. This is why we do not claim for any clone production. Still, in nuclear transfer experiments led in loach and goldfish on non enucleated oocytes, 60-70% of the reconstructed embryos were diploid [5,40]. Lower rates of 10-30% spontaneous enucleation were still observed in medaka [41]. Embryos were shown to be from donor nucleus origin although the mechanisms responsible for spontaneous maternal genome erasure are still to be investigated. Therefore, although the suitability of the injection conditions defined in this study must be tested on complete nuclear transfer experiments which would include enucleation, we are confident that they can also help to improve the nuclear transfer experiments led on non enucleated oocytes.

### Location of cell injection and cell incubation time before oocyte activation

Our results demonstrated that when the cell was injected too deep into the oocyte, the developments rates were low and the embryos died after the mid-blastula stage whereas injections higher toward the animal pole induced better development rates. We believe that in the case of deep injection, the nucleus was in an unsuitable environment. Indeed, during oogenesis, a polarity is established which determines the animal and vegetal pole, and maternal factors are unevenly distributed along this axis (reviewed by [42]). Our results suggest that it was essential for the donor chromatin to be exposed to specific animal pole factors. This hypothesis is reinforced by our observation that when the cell was injected at the right location but that the oocyte was activated without delay, the embryos also showed a reduced ability to develop beyond the mid-blastula stage. In this case, the chromatin was likely exposed to the appropriate ooplasmic reprogramming factors, but exposure time was too short to sustain reprogramming. This reprogramming hypothesis needs to be further investigated using even longer exposure time prior to activation, although in this case, the control of oocyte ageing will become a critical issue.

## Conclusions

The present work demonstrated that manipulation of metaphase II oocytes was possible in goldfish using trout coelomic fluid as an inactivating media. The toxicity of the medium injected into the oocyte proved to be a critical factor for oocyte ability to sustain development, and development rates were altered when large volumes were injected. The injection procedure through the micropyle allowed the nucleus to be positioned close to the animal pole, and we showed that deeper location was unsuitable for embryo development. The results were obtained on model systems including fertilized eggs and non enucleated oocytes. Several important steps of the procedure for somatic cell nuclear transfer were standardized in these conditions, and their application to clone production is expected to improve the development rates of the reconstructed embryos.

## Methods

### Gamete collection

Goldfish were from the U3E strain. Two years old fish raised in outdoor ponds were transferred into 0.3 m^3 ^tanks and reared several weeks in recycled water at 14°C under spring photoperiod. Fish were fed with carp pellets at 1% body weight. Three days before gamete collection, fish were transferred into 20°C water. Gamete release was stimulated by one injection of 0.5 mL/kg Ovaprim™ (synthetic salmonid GnRH with dopaminergic inhibitor, Syndel LTD, Canada) and gametes were collected 16 hours after injection. Spawns with homogeneous eggs displaying a rapid and neat blastodisc formation upon activation in water were kept for experiments. Fish handling and sampling was carried out in strict accordance with the welfare guiding principles of the French regulation on laboratory animals, under the supervision of staff possessing the highest agreement level (level 1 DSV).

### Embryo development and calculation of development rate

Spawn quality control was made by fertilizing 200 eggs with 10 μL sperm pool from 2 to 3 males in 10 mL tap water formerly aerated to remove HClO_4_. In all experiments, embryos obtained after fertilization and after nuclear transfer were incubated in tap water at 20°C. Development rate was recorded at 5 h (mid-blastula stage), 24 h (6-9 somites), and at hatching.

In a sample series of 33 spawns (150-200 eggs per spawn), rates for spawn quality controls (percentage of live embryo number to the total initial egg number) were 100% at 5 h, 88% ± 10 (min 62% - max 98%) at 24 h, and 81.2% ± 11.7 (min 50% - max 95%) at hatching. Because of this variability, development percentages after egg treatments were always expressed as a percentage of the corresponding spawn quality control.

### Selection of the medium enabling the prevention of oocyte activation

TCF was collected on spawns from freshly ovulated rainbow trout (*Oncorhynchus mykiss*) reared at the INRA Peima experimental farm. After sieving from the oocytes, TCF were centrifuged 30 min at 3600 g at 4°C and stored at -20°C before use. Soybean Trypsin Inhibitor (STI, Type II-S) solution with 0.1 mg/mL to 1 mg/mL STI was prepared in goldfish ringer (GFR: NaCl 125 mM, CaCl_2 _2H_2_O 2.4 mM, KCl 2.4 mM, MgSO_4 _7H_2_O 0.3 mM, MgCl_2 _6 H_2_O 0.9 mM, D glucose 6 mM, Hepes 4 mM, pH 7.3, 256 mOsm/kg). BSA (fraction V) solution at 5 mg/mL final concentration was prepared with STI 0.1 mg/mL in GFR. Synthetic ovarian fluid (SOF) was prepared according to [43]. For each spawn, fractions of 150-200 oocytes were incubated in 2 mL of the tested medium at 20°C for 30 min in plastic dish.

Prevention of oocyte activation in the tested medium meant that no cortical reaction was induced. Efficiency of the incubation medium was then deduced from the maintenance of oocyte ability to be fertilized. After incubation, the medium was removed by aspiration and oocytes were fertilized in tap water. The fertilization rate of the treated oocytes was recorded after 24 h development at 20°C. This stage cumulated most of the early developmental defects.

### Selection of the carrier medium

Toxicity of the medium injected into the oocyte with the donor cell during nuclear transfer was estimated in fertilization experiments. Oocytes were treated as in the nuclear transfer procedure, except that no donor cell was injected with the medium and that the treated oocytes were fertilized afterwards. Injected medium toxicity was deduced from the reduced ability of fertilized embryos to develop after medium injection into the oocytes. Twenty five to 50 oocytes were treated in each test, and the experiment was repeated on 3 to 6 different spawns. The media tested were cell culture medium (L-15 with Hepes 25 mM, NaHCO_3 _5 mM, 2 mM L-Glutamine, 2.5 μg/mL, and 5% fetal calf serum), phosphate buffer saline (PBS, Sigma France), SOF, goldfish coelomic fluid (GCF prepared as TCF), TCF, and TCF with 1 U/mL penicillin and 1 μg/mL streptomycin. All media contained 10% (v) phenol red 5 mg/mL (0.5 mg/mL final) to control that some medium is present in the oocytes after injection. The injected volume was about 50 pL. In control experiments, oocytes were punctured but no medium was injected.

### Donor cell and nuclear transfer

**Figure 6:**
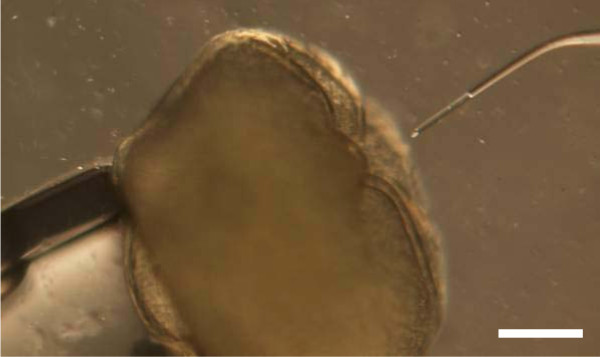
**Nuclear transfer through the micropyle of non activated oocytes**. The micropyle is seen as a large circular hole with hills and valley-shaped rim. Scale bar = 500 μm.

Donor epithelial cells [44] were obtained from caudal fin explant culture [2] and used after cell cryopreservation [1]. After thawing, cells were washed with cell culture medium with antibiotics (2.5 μg/mL amphotericin B, 50 μg/mL gentamicin) and stored on ice for up to 2 hours. Nuclear transfer was performed at 20°C using a Cell Tram Vario injector (Eppendorf, France) connected to a micromanipulator (Transferman NK2, Eppendorf, France) under a stereomicroscope (Olympus SZX 12). Recipient oocytes at the metaphase II stage were layered in a drop (1 mL) of TCF in a 10-cm plastic dish. Donor cells (30 μL) were spread in the TCF drop around the oocytes. Prior to donor cell injection, one recipient oocyte was positioned against a holding microcapillary (iD 100 μm). A single donor cell was aspirated in a glass microcapillary (iD 15 μm, Custom Tip Type IV, Eppendorf, France). Cell was transferred into the recipient oocyte through the micropyle at the animal pole (Figure 6) according to [6,8,15]. After the 20 oocytes were injected (15-20 min), oocytes were activated with tap water and incubated at 20°C for embryo development. Control oocytes maintained in TCF but not injected were fertilized and assessed for development control.

### Optimization of the injection procedure

Two depths at which donor cell was injected were tested. Oocytes were treated as in the nuclear transfer procedure. In one case, the opening of the microcapillary containing donor cell was pushed up to center of the oocyte and the donor cell was injected. In the second case, the opening of the microcapillary was positioned inside the oocyte close to the plasma membrane by a slight outward movement after capillary penetration into the oocyte, and the donor cell was injected. Forty oocytes were treated in each case, and the experiment was repeated on 7 different spawns.

The incubation time before oocytes were activated after nuclear transfer was tested. Oocytes were treated as in the nuclear transfer procedure. After injection, oocytes were immediately activated, or incubated in TCF at 20°C for 30 and 60 min before activation. Forty oocytes were treated in each case, and the experiment was repeated on 4 to 6 different spawns.

### Recording of membrane rupture upon injection

Membrane rupture of donor cells prior to injection was not possible with fin cultured cells in our conditions. Mechanical forces (several aspiration and expulsion through the microcapillary) and osmotic shocks (up to 0 mOsm/Kg) were tested, but unsuccessfully. No other treatments such as nitrogen cavitation or mild digestion by trypsin, lysolecithin, or triton improved membrane rupture protocol either. Therefore, the whole cell was injected into the oocyte. The behavior of the cell once injected in the oocyte was assessed in two nuclear transfer experiments. Prior to transfer, donor cells were labeled in Calcein AM 2 μM (Molecular Probes) in PBS for 30 min at room temperature. Labeled cell were then transferred in TCF on the nuclear transfer stage and transferred into the oocytes as described above. The delay between cell injection and membrane rupture was assessed under fluorescence with the stereomicroscope used for nuclear transfer.

### Statistics

Results are expressed as the mean percentages ± SE. Statistical significance of differences between development percentages was determined by the distribution-free U test of Mann-Whitney using STATISTICA^® ^software (StatSoft^®^).

## Authors' contributions

GM initiated the project in the group. PYLB and CL conceived and designed the study and coordinated the experiments. SM threw the difficult methodological basis of our first nuclear transfer experiments. AD carried out the nuclear transfer experiments and fin cell culture and cryopreservation. NC initiated the first trials on metaphase II oocytes and designed the inactivation media experiments. PYLB, AD and CL analyzed the data. CL wrote the paper. All authors read and approved the final manuscript.

## References

[B1] Mauger PE Le Bail PY Labbe C Cryobanking of fish somatic cells: optimizations of fin explant culture and fin cell cryopreservation Comp Biochem Physiol B: Biochem Mol Biol 2006 144 29 37 10.1016/j.cbpb.2006.01.004 16503180

[B2] Moritz C Labbe C Cryopreservation of goldfish fins and optimization for field scale cryobanking Cryobiology 2008 56 181 188 10.1016/j.cryobiol.2008.02.003 18346725

[B3] Robles V Cabrita E Herraez MP Germplasm cryobanking in zebrafish and other aquarium model species Zebrafish 2009 6 281 293 10.1089/zeb.2009.0592 19761380

[B4] Sun YH Chen SP Wang YP Hu W Zhu ZY Cytoplasmic impact on cross-genus cloned fish derived from transgenic common carp (Cyprinus carpio) nuclei and goldfish (Carassius auratus) enucleated eggs Biol Reprod 2005 72 510 515 10.1095/biolreprod.104.031302 15469998

[B5] Gasaryan KG Hung NM Neyfakh AA Ivanenkov VV Nuclear transplantation in teleost Misgurnus fossilis L Nature 1979 280 585 587 10.1038/280585a0 572485

[B6] Niwa K Ladygina T Kinoshita M Ozato K Wakamatsu Y Transplantation of blastula nuclei to non-enucleated eggs in the medaka, Oryzias latipes Dev Growth Differ 1999 41 163 172 10.1046/j.1440-169x.1999.00423.x 10223712

[B7] Hongtuo F Chingjiang W Nuclear transfer in loach (Paramisgurnus dabryanus Sauvage) by cell-to-cell electrofusion Aquacult Res 2001 32 267 275 10.1046/j.1365-2109.2001.00555.x

[B8] Wakamatsu Y Ju B Pristyaznhyuk I Niwa K Ladygina T Kinoshita M Araki K Ozato K Fertile and diploid nuclear transplants derived from embryonic cells of a small laboratory fish, medaka (Oryzias latipes) Proc Natl Acad Sci USA 2001 98 1071 1076 10.1073/pnas.98.3.1071 11158596 PMC14710

[B9] Lee KY Huang H Ju B Yang Z Lin S Cloned zebrafish by nuclear transfer from long-term-cultured cells Nat Biotechnol 2002 20 795 799 12134167 10.1038/nbt721

[B10] Huang H Ju B Lee KY Lin S Protocol for nuclear transfer in zebrafish Cloning Stem Cells 2003 5 333 337 10.1089/153623003772032835 14733751

[B11] Ju B Huang H Lee KY Lin S Cloning zebrafish by nuclear transfer Methods Cell Biol 2004 77 403 411 full_text 15602924 10.1016/s0091-679x(04)77022-3

[B12] Kaftanovskaya E Motosugi N Kinoshita M Ozato K Wakamatsu Y Ploidy mosaicism in well-developed nuclear transplants produced by transfer of adult somatic cell nuclei to nonenucleated eggs of medaka (Oryzias latipes) Dev Growth Differ 2007 49 691 698 17868281 10.1111/j.1440-169X.2007.00962.x

[B13] Wakamatsu Y Novel method for the nuclear transfer of adult somatic cells in medaka fish (Oryzias latipes): use of diploidized eggs as recipients Dev Growth Differ 2008 50 427 436 10.1111/j.1440-169X.2008.01050.x 18638166

[B14] Siripattarapravat K Busta A Steibel JP Cibelli J Characterization and in vitro control of MPF activity in zebrafish eggs Zebrafish 2009 6 97 105 10.1089/zeb.2008.0527 19292671

[B15] Siripattarapravat K Pinmee B Venta PJ Chang CC Cibelli JB Somatic cell nuclear transfer in zebrafish Nat Methods 2009 6 733 735 10.1038/nmeth.1369 19718031

[B16] Vignon X Chesne P Le Bourhis D Flechon JE Heyman Y Renard JP Developmental potential of bovine embryos reconstructed from enucleated matured oocytes fused with cultured somatic cells C R Acad Sci Paris- Series III - Sci Vie 1998 321 735 745 10.1016/s0764-4469(98)80014-0 9809205

[B17] Verma PJ Du ZT Crocker L Faast R Grupen CG McIlfatrick SM Ashman RJ Lyons IG Nottle MB In vitro development of porcine nuclear transfer embryos constructed using fetal fibroblasts Mol Reprod Dev 2000 57 262 269 10.1002/1098-2795(200011)57:3<262::AID-MRD8>3.0.CO;2-X 11013434

[B18] Wilmut I Schnieke AE McWhir J Kind AJ Campbell KH Viable offspring derived from fetal and adult mammalian cells Nature 1997 385 810 813 10.1038/385810a0 9039911

[B19] Baguisi A Behboodi E Melican DT Pollock JS Destrempes MM Cammuso C Williams JL Nims SD Porter CA Midura P Palacios MJ Ayres SL Denniston RS Hayes ML Ziomek CA Meade HM Godke RA Gavin WG Overstrom EW Echelard Y Production of goats by somatic cell nuclear transfer Nat Biotechnol 1999 17 456 461 10.1038/8632 10331804

[B20] Choi YH Love CC Chung YG Varner DD Westhusin ME Burghardt RC Hinrichs K Production of nuclear transfer horse embryos by piezo-driven injection of somatic cell nuclei and activation with stallion sperm cytosolic extract Biol Reprod 2002 67 561 567 10.1095/biolreprod67.2.561 12135896

[B21] Wakayama T Perry ACF Zuccotti M Johnson KR Yanagimachi R Full-term development of mice from enucleated oocytes injected with cumulus cell nuclei Nature 1998 394 369 374 10.1038/28615 9690471

[B22] Niwa K Kani S Kinoshita M Ozato K Wakamatsu Y Expression of GFP in nuclear transplants generated by transplantation of embryonic cell nuclei from GFP-transgenic fish into nonenucleated eggs of medaka, Oryzias latipes Cloning 2000 2 23 34 10.1089/15204550050145102 16218843

[B23] Ju B Pristyazhnyuk I Ladygina T Kinoshita M Ozato K Wakamatsu Y Development and gene expression of nuclear transplants generated by transplantation of cultured cell nuclei into non-enucleated eggs in the medaka Oryzias latipes Dev Growth Differ 2003 45 167 174 10.1034/j.1600-0854.2004.00687.x 12752504

[B24] Bubenshchikova E Kaftanovskaya E Motosugi N Fujimoto T Arai K Kinoshita M Hashimoto H Ozato K Wakamatsu Y Diploidized eggs reprogram adult somatic cell nuclei to pluripotency in nuclear transfer in medaka fish (Oryzias latipes) Dev Growth Differ 2007 49 699 709 17868280 10.1111/j.1440-169X.2007.00963.x

[B25] Fernandez J Valladares M Fuentes R Ubilla A Reorganization of cytoplasm in the zebrafish oocyte and egg during early steps of ooplasmic segregation Dev Dyn 2006 235 656 671 10.1002/dvdy.20682 16425221

[B26] Mansouri-Attia Nr Sandra O Aubert J Degrelle S+ Everts RE Giraud-Delville C Heyman Y Galio L Hue I Yang X Tian XC Lewin HA Renard JP Endometrium as an early sensor of in vitro embryo manipulation technologies Proc Natl Acad Sci USA 2009 106 5687 5692 10.1073/pnas.0812722106 19297625 PMC2667091

[B27] Hsu S-Y Goetz FW Inhibition of chorion expansion and preservation of fertility in golfish (*Carassius auratus*) eggs by protease inhibitors Can J Fish Aquat Sci 1993 50 932 935 10.1139/f93-107

[B28] Coffman MA Goetz FW Trout ovulatory proteins are partially responsible for the anti-proteolytic activity found in trout coelomic fluid Biol Reprod 1998 59 497 502 10.1095/biolreprod59.3.497 9716546

[B29] Lahnsteiner F Weismann T Patzner RA Composition of the ovarian fluid in 4 salmonid species: Oncorhynchus mykiss, Salmo trutta f lacustris, Salvelinus alpinus and Hucho hucho Reprod Nutr Dev 1995 35 465 474 10.1051/rnd:19950501 8526977

[B30] Coffman MA Pinter JH Goetz FW Trout ovulatory proteins: site of synthesis, regulation, and possible biological function Biol Reprod 2000 62 928 938 10.1095/biolreprod62.4.928 10727262

[B31] Formacion MJ Venkatesh B Tan CH Lam TJ Overripening of Ovulated Eggs in Goldfish, Carassius-Auratus 2. Possible Involvement of Postovulatory Follicles and Steroids Fish Physiol Biochem 1995 14 237 246 10.1007/BF00004314 24197445

[B32] Chenais N Depince A Le Bail PY Labbe C Variation in egg quality after hormonally-induced ovulation in goldfish is more related to female variability than to short term post-ovulation ageing Cybium 2008 32 236

[B33] Rime H Guitton N Pineau C Bonnet E Bobe J Jalabert B Post-ovulatory ageing and egg quality: A proteomic analysis of rainbow trout coelomic fluid BMC Reprod Biol Endocrinol 2004 2 1 10 10.1186/1477-7827-2-1 PMC443514 15180895

[B34] Lee KW Webb SE Miller AL A wave of free cytosolic calcium traverses zebrafish eggs on activation Dev Biol 1999 214 168 180 10.1006/dbio.1999.9396 10491266

[B35] Leung CF Webb SE Miller AL Calcium transients accompany ooplasmic segregation in zebrafish embryos Dev Growth Differ 1998 40 313 326 10.1046/j.1440-169X.1998.t01-1-00007.x 9639359

[B36] Webb SE Lee KW Karplus E Miller AL Localized calcium transients accompany furrow positioning, propagation, and deepening during the early cleavage period of zebrafish embryos Dev Biol 1997 192 78 92 10.1006/dbio.1997.8724 9405098

[B37] Poccia D Collas P Transforming sperm nuclei into male pronuclei *in vivo *and *in vitro* Curr Topics Dev Biol 1996 34 25 88 full_text 10.1016/s0070-2153(08)60708-5 8787571

[B38] Guttinger S Laurell E Kutay U Orchestrating nuclear envelope disassembly and reassembly during mitosis Nat Rev Mol Cell Biol 2009 10 178 191 10.1038/nrm2641 19234477

[B39] Yan SY Tu M Yang HY Mao ZG Zhao ZY Fu LJ Li GS Huang GP Li SH Jin GQ Developmental incompatibility between cell nucleus and cytoplasm as revealed by nuclear transplantation experiments in teleost of different families and orders Int J Dev Biol 1990 34 255 266 2386727

[B40] Liu TM Yu XM Ye YZ Zhou JF Wang ZW Tong JG Wu CJ Factors affecting the efficiency of somatic cell nuclear transplantation in the fish embryo J Exp Zool 2002 293 719 725 10.1002/jez.10177 12410600

[B41] Bubenshchikova E Ju B Pristyazhnyuk I Niwa K Kaftanovskaya E Kinoshita M Ozato K Wakamatsu Y Generation of fertile and diploid fish, medaka (Oryzias latipes), from nuclear transplantation of blastula and four-somite-stage embryonic cells into nonenucleated unfertilized eggs Cloning Stem Cells 2005 7 255 264 10.1089/clo.2005.7.255 16390261

[B42] Pelegri F Maternal factors in zebrafish development Dev Dyn 2003 228 535 554 10.1002/dvdy.10390 14579391

[B43] Sun Y Zhang C Liu S Duan W Liu Y Induced interspecific androgenesis using diploid sperm from allotetraploid hybrids of common carp×red crucian carp Aquaculture 2007 264 47 53 10.1016/j.aquaculture.2006.07.004

[B44] Mauger PE Labbe C Bobe J Cauty C Leguen I Baffet G Le Bail PY Characterization of goldfish fin cells in culture: some evidence of an epithelial cell profile Comp Biochem Physiol B Biochem Mol Biol 2009 152 205 215 10.1016/j.cbpb.2008.11.003 19068235

